# Joint Client Selection and CPU Frequency Control in Wireless Federated Learning Networks with Power Constraints

**DOI:** 10.3390/e25081183

**Published:** 2023-08-09

**Authors:** Zhaohui Zhou, Shijie Shi, Fasong Wang, Yanbin Zhang, Yitong Li

**Affiliations:** School of Electrical and Information Engineering, Zhengzhou University, Zhengzhou 450001, China

**Keywords:** federated learning, client selection, CPU frequency control, wireless networks

## Abstract

Federated learning (FL) represents a distributed machine learning approach that eliminates the necessity of transmitting privacy-sensitive local training samples. However, within wireless FL networks, resource heterogeneity introduces straggler clients, thereby decelerating the learning process. Additionally, the learning process is further slowed due to the non-independent and identically distributed (non-IID) nature of local training samples. Coupled with resource constraints during the learning process, there arises an imperative need for optimizing client selection and resource allocation strategies to mitigate these challenges. While numerous studies have made strides in this regard, few have considered the joint optimization of client selection and computational power (i.e., CPU frequency) for both clients and the edge server during each global iteration. In this paper, we initially define a cost function encompassing learning latency and non-IID characteristics. Subsequently, we pose a joint client selection and CPU frequency control problem that minimizes the time-averaged cost function subject to long-term power constraints. By utilizing Lyapunov optimization theory, the long-term optimization problem is transformed into a sequence of short-term problems. Finally, an algorithm is proposed to determine the optimal client selection decision and corresponding optimal CPU frequency for both the selected clients and the server. Theoretical analysis provides performance guarantees and our simulation results substantiate that our proposed algorithm outperforms comparative algorithms in terms of test accuracy while maintaining low power consumption.

## 1. Introduction

As the rapid expansion of Internet of Things (IoT) communication unfolds, an immense volume of data generated by massive machine-type devices circulates via wireless access technology. This advancement instigates the pervasive utilization of machine-learning-based applications in various aspects of people’s everyday life, including smart transportation and smart healthcare. In the traditional centralized learning paradigm, the raw data of each device are initially uploaded to the edge server via a wireless channel, facilitating aggregation for subsequent processing and analysis. This methodology, however, potentially leads to privacy data leakage. Consequently, the exploration of machine learning mechanisms that protect user data privacy is of paramount significance. FL was proposed as a solution to address the limitations of traditional centralized machine learning methods in ensuring user data privacy [1]. This approach permits each device to partake in the collaborative training of a shared model, negating the need to share the device’s proprietary data. Since its inception, federated learning has garnered considerable interest from both academia and industry, finding broad applications in fields such as mobile cloud computing [2], the industrial Internet of Things [3], and device-to-device communication [4].

Despite the significant advantages offered by federated learning, its application in wireless networks presents certain challenges that warrant attention. Firstly, the scarcity of wireless resources, such as channel bandwidth, limit the number of clients capable of participating in each learning iteration. Additionally, client selection for each iteration needs to be guided by the real-time state of the channel. Secondly, the computational power and power budget of devices and the edge server are finite, necessitating the optimization of computational power and power allocation throughout the multi-iteration learning process in FL, which is essential for both extending battery life and advancing green communication. A third challenge arises from the latency incurred during the federated learning process, which constrains its use in scenarios that are latency-sensitive. The latency is determined by the time required for straggler clients to upload the local model during each learning iteration, the time spent aggregating the model on the edge server, which is influenced by the transmission power and CPU frequency in each learning iteration. Furthermore, the heterogeneity of client behavior and the variable dynamics of wireless environments may lead to the acquisition of non-independent and identically distributed (non-IID) training data [5]. Some clients may hold data that significantly deviate from independent identically distributed (IID) training data, making it challenging for the model to generalize effectively across all clients.

### 1.1. Related Work

Ever since the proposition of federated learning, an extensive research effort has been devoted to improving its performance in wireless networks. This advancement primarily hinges on designing appropriate client selection schemes [6,7] or optimizing resource allocation [8,9,10]. For instance, a client scheduling strategy based on channel and learning qualities was proposed in [7]. Ref. [9] investigated both the CPU frequency and transmission power control strategy of all IoT clients to minimize the energy consumption under latency requirement. The studies presented in [11,12,13] focused on the optimization of the client selection process and resource allocation simultaneously, with the objective of improving federated learning performance. Specifically, Ref. [11] delved into a joint client selection and resource allocation problem, seeking to optimize the trade-off between the number of selected clients and the total energy consumption, while [12] focused on minimizing training loss while adhering to the constraints of delay and energy consumption. Additionally, Ref. [13] explored the process of client selection and resource allocation under the condition of non-IID data distributions.

The aforementioned studies were formulated in the context of individual global iterations, overlooking the interdependence between different iterations. This neglects the cumulative learning effect over multiple iterations, potentially yielding less effective learning models and limiting overall system performance. Hence, the need for long-term optimization that accounts for the interconnectedness of global iterations becomes evident. Numerous research efforts [14,15,16,17,18] have targeted long-term optimization in federated learning, focusing on various aspects of the problem. For instance, Ref. [14] sought to optimize the client selection process in each learning round with the aim of minimizing training latency under fairness constraints. The study presented in [15] introduced a dynamic scheduling mechanism aimed at optimizing the federated learning process, striking a balance between the enhancement of learning performance and the reduction of training latency. Ref. [16] focused on the optimization of radio transmission parameters and computation resources, attempting to minimize power consumption while upholding learning performance and latency constraints. Refs. [17,18] focused on client selection and bandwidth allocation under energy constraints in wireless FL networks. Specifically, the study in [17] aimed to maximize the weighted sum of selected clients, whereas [18] focused on minimizing the cost of time and accuracy. While the above works explored long-term optimization in federated learning, the optimization of the latency and the impact of its non-IID nature under the long-term power constraints of both clients and the server for FL have not been considered.

### 1.2. Contribution

In this paper, we consider a client selection and CPU frequency control problem in wireless FL networks. Different from the extant literature, our approach concurrently optimizes the selection of clients and CPU frequency for both clients and the edge server. The objective of the proposed problem is to minimize a predefined cost function, which incorporates latency and model robustness, under long-term power constraints. The main contributions of our work are as follows:

(1) We develop a comprehensive framework for the long-term client selection and CPU frequency control problem, taking into account the interdependence of different global iterations and long-term power consumption constraints for both clients and the server. The aim is to expedite the learning process by incorporating client and server latency, as well as the effect of the non-IID distribution of local training samples.
(2) Leveraging Lyapunov optimization theory, we transform the long-term problem into a set of per-iteration problems. We introduce an algorithm to tackle the per-iteration problem, accompanied by a theoretical performance guarantee.
(3) We conduct extensive experiments, inclusive of several comparative experiments. Simulation results demonstrate that our proposed algorithm can yield superior test accuracy while maintaining low power consumption.

The remainder of this paper is structured as follows. Our proposed framework’s system model, along with the optimization problem formulation, is elucidated in Section 2. The solution via the Lyapunov optimization theory, is laid out in Section 3. Section 4 comprises the simulation results, showcasing the superiority of our proposed scheme. Finally, Section 5 concludes and discusses the paper.

## 2. System Model and Problem Formulation

**Figure 1:**
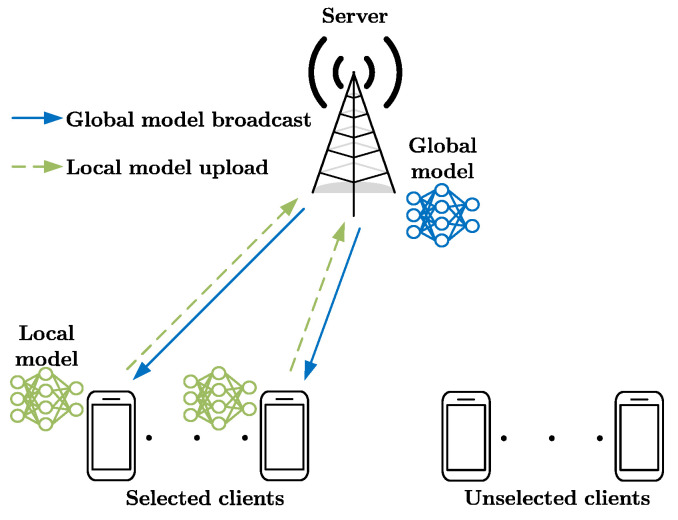
Federated learning framework in wireless networks.

The proposed federated learning framework is shown in Figure 1, consisting of a set of clients K={1,…,K} and a server, with *K* indicating the total number of clients. Each client k∈K possesses a local dataset $D_{k}={\left\{\left(x_{i},y_{i}\right)\right\}}_{i=1}^{d_{k}}$, wherein $x_{i}$ and $y_{i}$ denote the *i*-th sample and its associated ground-truth label of client *k*, respectively, and $d_{k}$ stands for the dataset size originating from $q_{k}$ label classes.

### 2.1. Learning Model

Assuming *T* global iterations, we adopt $a_{k}^{t}=1$ to represent the selection of client *k* in global iteration t=0,…,T−1, with $a_{k}^{t}=0$ otherwise. The client selection decisions are denoted by $a^{t}=\left(a_{1}^{t},\ldots ,a_{K}^{t}\right)$. The server aims to construct a global model by minimizing the following global loss function:

(1) $$w^{*}=\underset{w}{\arg\min}\frac{\sum_{k=1}^{K}\left[d_{k}f_{k}\left(w\right)\right]}{\sum_{k=1}^{K}d_{k}},$$

where $f_{k}\left(w\right)$ is the local loss function at client *k*. For instance, the loss function for linear regression is given by:

(2) $$f_{k}\left(w\right)=\frac{1}{2}{\left(x_{i}^{T}w-y_{i}\right)}^{2}.$$

The goal of the training process is to find the optimal model $w^{*}$ though iteration. A global iteration *t* consists of four steps:

(1) Each client shares its side’s information. Subsequently, the server selects a group of clients and broadcasts the current global model $w^{t}$ to them.
(2) The selected clients execute a local iteration to update their local models $w_{k}^{t}$ based on their respective datasets.
(3) The selected clients upload their newly updated local models to the server.
(4) The server aggregates all the received local models to establish a new global model, as represented by $w^{t+1}=\sum_{k=1}^{K}\left(a_{k}^{t}w_{k}^{t}\right)/\sum_{k=1}^{K}a_{k}^{t}$.

### 2.2. Power Consumption Model

In each global iteration, the selected clients engage in training and uploading models while the server aggregates these received models. This process contributes to power consumption. We represent the overall CPU frequency control decisions of the clients as $f^{t}=\left(f_{1}^{t},\ldots ,f_{K}^{t}\right)$, where $f_{k}^{t}$ indicates the CPU frequency of client *k* in the global iteration *t*. Notably, if $a_{k}^{t}=0$, then $f_{k}^{t}$ also equals zero. The power computation of the training model can be expressed as $P_{k}^{t,tr}=\gamma_{1}{\left(f_{k}^{t}\right)}^{3}a_{k}^{t}$, where $\gamma_{1}$ denotes the capacitance coefficient of clients [18]. Let $P_{k}^{t,up}=p_{k}^{t}a_{k}^{t}$ denote the power spent for uploading the model; thus, the total power consumption of client *k* during the global iteration *t* is given by:

(3) $$P_{k}^{t}=P_{k}^{t,tr}+P_{k}^{t,up}.$$

On the other hand, the CPU frequency of the server during the global iteration *t* is represented by $f_{r}^{t}$, and the server’s capacitance coefficient is denoted as $\gamma_{2}$. Consequently, the power consumption of the server during the global iteration *t* can be formulated as follows:

(4) $$P_{r}^{t}=\gamma_{2}{\left(f_{r}^{t}\right)}^{3}.$$

### 2.3. Latency Model

Let *m* represent the number of local iterations in each global iteration, and $c_{k}$ stand for the number of CPU cycles necessary to process a sample from client *k*. The local training latency for selected client *k* in global iteration *t* can then be calculated as $\tau_{k}^{t,tr}=mc_{k}d_{k}a_{k}^{t}/f_{k}^{t}$, which will linearly decrease as the allocated local computing power $f_{k}^{t}$ increases.

When the local training is finished, the selected clients upload their models to the server via orthogonal frequency-division multiple access (OFDMA). The total available bandwidth is denoted as *B*, and it is assumed that this bandwidth is equally allocated to the selected clients during the global iteration *t*. Consequently, the bandwidth allocated to a selected client *k* in global iteration *t* can be represented as

(5) $$b^{t}=B/\sum_{k=1}^{K}a_{k}^{t}.$$

The model size is represented as *s*; therefore, the latency for model uploading is given by

(6) $$\tau_{k}^{t,up}=\frac{sa_{k}^{t}}{b^{t}\log_{2}\left(1+\frac{h_{k}^{t}p_{k}^{t}}{N_{0}b^{t}}\right)},$$

where $h_{k}^{t}$ denotes the channel gain between client *k* and the server during the global iteration *t*, which is assumed to be available at the transmitter side. $N_{0}$ denotes the power spectral density of noise. The total latency of client *k* can be formulated as:

(7) $$\tau_{k}^{t}=\tau_{k}^{t,tr}+\tau_{k}^{t,up}.$$

At the server side, let $\tau_{r}^{t}$ denote the latency of the server in global iteration *t*, which can be written as:

(8) $$\tau_{r}^{t}=\frac{\phi \sum_{k=1}^{K}a_{k}^{t}}{f_{r}^{t}},$$

where ϕ is the quantity of processing cycles required to carry out a single summation operation [16].

We assume that the server starts aggregating after receiving all the local models of selected clients. Therefore, the learning latency of global iteration *t* is bottlenecked by the straggler clients and can be derived as:

(9) $$\tau^{t}=\max_{k=1,\ldots K}\left(\tau_{k}^{t}\right)+\tau_{r}^{t}.$$

### 2.4. Cost Model

The non-IID nature of data introduces biases in the training process, which significantly impacts the accuracy of FL. As noted in [13], a larger number of label classes might result in a more robust trained model, and the non-IID nature could decrease when clients possess more label classes. In this paper, we use label classes $q_{k}$ to quantify the non-IID nature with an aim to minimize both the learning latency and accuracy degradation caused by it. However, reducing the latter could potentially increase the learning latency. Therefore, we propose a cost objective function $U^{t}$ to balance the two goals during the global iteration *t*:

(10) $$U^{t}\left(a^{t},f^{t},f_{r}^{t}\right)=\tau^{t}-\mu \sum_{k=1}^{K}\left(a_{k}^{t}q_{k}\right),$$

where μ is a price parameter, which turns the label classes into a cost form [19].

### 2.5. Problem Formulation

From the aforementioned discussion, we consider an optimization problem that minimizes the time-averaged cost function through joint client selection and CPU frequency control as follows:

(11) $$\begin{array}{cc} P1\; & \min_{G^{0},\ldots ,G^{T-1}}\frac{1}{T}\sum_{t=0}^{T-1}U^{t}\left(a^{t},f^{t},f_{r}^{t}\right) \end{array}$$

(12) $$\begin{array}{cc} \; & s.t.\;f_{k}^{\min}\leq f_{k}^{t}\leq f_{k}^{\max},\forall k,\forall t, \end{array}$$

(13) $$\begin{array}{cc} \; & \;f_{r}^{\min}\leq f_{r}^{t}\leq f_{r}^{\max},\forall t, \end{array}$$

(14) $$\begin{array}{cc} \; & \;a_{k}^{t}\in \left\{0,1\right\},\forall k,\forall t, \end{array}$$

(15) $$\begin{array}{cc} \; & \;\frac{1}{T}\sum_{t=0}^{T-1}P_{k}^{t}\leq {\bar{P}}_{k},\forall k, \end{array}$$

(16) $$\begin{array}{cc} \; & \;\frac{1}{T}\sum_{t=0}^{T-1}P_{r}^{t}\leq {\bar{P}}_{r}. \end{array}$$

where $G^{t}=\left(a^{t},f^{t},f_{r}^{t}\right)$ is the optimization variables in global iteration *t*, t=0,1,…,T−1. Constraint (12) and (13) specify the CPU frequency range of each client and the server, respectively. Constraint (14) defines whether each client is selected or not. Constraint (15) guarantees that the average power consumption of each client is limited by ${\bar{P}}_{k}$, while constraint (16) guarantees that the average power consumption of the server is limited by ${\bar{P}}_{r}$. For clarity, in the following sections of this paper, we succinctly refer to the cost function introduced in Equation (10) as $U^{t}$.

## 3. Problem Solution and Algorithm Design

A direct resolution of problem P1 is not viable due to the time-averaged optimization objective and long-term power constraints. Therefore, in this paper, problem P1 is initially transformed into a per-iteration problem by utilizing Lyapunov optimization theory. Subsequently, this per-iteration problem is decomposed into two distinct subproblems: a CPU frequency control problem, which assumes fixed client selection decisions, and a client selection problem that operates under the optimal CPU frequency setting.

### 3.1. Problem Transformation via Stochastic Optimization Theory

The resolution of problem P1 necessitates comprehensive information, such as channel gain, pertaining to *T* global iterations. However, the unavailability of future information in the present moment presents a formidable challenge. To circumvent this issue, P1 is converted into a series of subproblems, the solutions for which do not rely on the knowledge of future iterations. This transformation is achieved through the application of Lyapunov optimization theory [20] and the introduction of virtual queue techniques. For each client, a virtual power deficit queue $Z_{k}^{t}$ is established, with an initial condition of $Z_{k}^{0}=0$, and updated at the end of each global iteration as follows:

(17) $$Z_{k}^{t+1}=\max\left\{P_{k}^{t}-{\bar{P}}_{k}+Z_{k}^{t},0\right\},$$

where $Z_{k}^{t}$ encapsulates the disparity between power consumption and the long-term power constraint of client *k* over *T* iterations. A similar approach can be used to construct a virtual power deficit queue $Y_{r}^{t}$ for the server, as depicted:

(18) $$Y_{r}^{t+1}=\max\left\{P_{r}^{t}-{\bar{P}}_{r}+Y_{r}^{t},0\right\}.$$

To maintain the mean rate stability of the queues, we first establish a Lyapunov function in the following form:

(19) $$L\left(\Theta^{t}\right)=\frac{1}{2}\left[\sum_{k=1}^{K}{\left(Z_{k}^{t}\right)}^{2}+{\left(Y_{r}^{t}\right)}^{2}\right],$$

where $\Theta^{t}$ symbolizes all the virtual deficit queues. Then we formulate Lyapunov drift to measure the expected increase as of $L(\Theta^{t})$ as follows:

(20) $$\Delta \left(\Theta^{t}\right)=E\left[L\left(\Theta^{t+1}\right)-L\left(\Theta^{t}\right)|\Theta^{t}\right].$$

With the objective of restricting the growth of virtual deficit queues and minimizing the cost function, the objective function is integrated into the Lyapunov drift. Consequently, the drift-plus-cost function is defined as follows:

(21) $$\Delta \left(\Theta^{t}\right)+VE\left[U^{t}|\Theta^{t}\right],$$

where *V* serves as a control parameter that aids in balancing the trade-off between minimizing the objective function and adhering to the power constraints. An observation of (21) indicates that it solely involves the current iteration *t*, signifying that the original problem P1 can be transitioned into a real-time problem solved on a per-iteration basis. The application of Lyapunov optimization theory provides the following lemma regarding the upper bound of the drift-plus-cost function:

**Theorem** **1.** *Assume $P_{k}^{\max}\geq P_{k}^{t}$ for each client k, and $P_{r}^{\max}\geq P_{r}^{t}$ for the server in global iteration t. The drift-plus-cost function satisfies:*

(22) $$\Delta \left(\Theta^{t}\right)+VE\left[U^{t}|\Theta^{t}\right]\leq C_{1}+\sum_{k=1}^{K}Z_{k}^{t}E\left[P_{k}^{t}-{\bar{P}}_{k}|\Theta^{t}\right]+Y_{r}^{t}E\left[P_{r}^{t}-{\bar{P}}_{r}|\Theta^{t}\right]+VE\left[U^{t}|\Theta^{t}\right],$$

*where $C_{1}$ is a finite constant, which satisfies $C_{1}\geq \frac{1}{2}\sum_{k=1}^{K}{\left(P_{k}^{\max}-{\bar{P}}_{k}\right)}^{2}+\frac{1}{2}{\left(P_{r}^{\max}-{\bar{P}}_{r}\right)}^{2}$.*

**Proof.**  The proof is given in Appendix A. □

By minimizing the upper bound in Equation (22), virtual deficit queue stability is achieved concurrently with cost function minimization. Upon excluding all constants (i.e., *C*, ${\bar{P}}_{k}Z_{k}^{t}$, ${\bar{P}}_{r}Y_{r}^{t}$), problem P1 can be transformed into a per-iteration problem P2:

(23) $$\begin{array}{cc} P2\; & \min_{G^{t}}\sum_{k=1}^{K}\left(P_{k}^{t}Z_{k}^{t}\right)+P_{r}^{t}Y_{r}^{t}+VU^{t}\\ \; & s.t.\;(12)–(14). \end{array}$$

### 3.2. Problem Solution

To simplify the complexity, $U^{t}$ in Equation (21) is substituted with an upper bound ${\tilde{U}}^{t}=\sum_{k=1}^{K}\tau_{k}^{t}+\tau_{r}^{t}-\mu \sum_{k=1}^{K}\left(a_{k}^{t}q_{k}^{t}\right)$, derivable through the application of $\max_{k=1,\ldots K}\left(\tau_{k}^{t}\right)\leq \sum_{k=1}^{K}\tau_{k}^{t}$. Consequently, the resolution of P2 can be reoriented towards the following problem:

(24) $$\begin{array}{cc} P3\; & \min_{G^{t}}\sum_{k=1}^{K}\left(P_{k}^{t}Z_{k}^{t}\right)+P_{r}^{t}Y_{r}^{t}+V{\tilde{U}}^{t}\\ \; & s.t.\;(12)–(14). \end{array}$$

Problem P3 manifests as a mixed-integer problem and poses a significant challenge for direct resolution. However, given any $a^{t}$, the objective function of P3 transforms into a convex function with respect to the CPU frequency of the selected clients and the server, i.e., $f_{k}^{t}$ and $f_{r}^{t}$. Consequently, the optimal CPU frequencies for selected clients and the server can be efficiently procured as

(25) $${\left(f_{k}^{t}\right)}^{*}=\left\{\begin{array}{c} f_{k}^{\min},if\sqrt[{4}]{\frac{Vmc_{k}d_{k}}{3Z_{k}^{t}\gamma_{1}}}<f_{k}^{\min}\\ f_{k}^{\max},if\sqrt[{4}]{\frac{Vmc_{k}d_{k}}{3Z_{k}^{t}\gamma_{1}}}>f_{k}^{\max}\\ \sqrt[{4}]{\frac{Vmc_{k}d_{k}}{3Z_{k}^{t}\gamma_{1}}},\mathrm{otherwise}, \end{array}\right.$$

and

(26) $${\left(f_{r}^{t}\right)}^{*}=\left\{\begin{array}{c} f_{r}^{\min},if\sqrt[{4}]{\frac{V\phi \sum_{k=1}^{K}a_{k}^{t}}{3Y_{r}^{t}\gamma_{2}}}<f_{r}^{\min}\\ f_{r}^{\max},if\sqrt[{4}]{\frac{V\phi \sum_{k=1}^{K}a_{k}^{t}}{3Y_{r}^{t}\gamma_{2}}}>f_{r}^{\max}\\ \sqrt[{4}]{\frac{V\phi \sum_{k=1}^{K}a_{k}^{t}}{3Y_{r}^{t}\gamma_{2}}},\mathrm{otherwise}, \end{array}\right.$$

respectively.

With the optimal CPU frequency established, the objective of problem P2 becomes a function of $a^{t}$ and can consequently be transformed as follows:

(27) $$\begin{array}{cc} P4\; & \min_{a^{t}}\sum_{k=1}^{K}\left(P_{k}^{t}Z_{k}^{t}\right)+P_{r}^{t}Y_{r}^{t}+VU^{t}\\ \; & s.t.\;f_{k}^{t}={\left(f_{k}^{t}\right)}^{*},\forall k,\\ \; & \;f_{r}^{t}={\left(f_{r}^{t}\right)}^{*},\\ \; & \;(14). \end{array}$$

A straightforward strategy to resolve P4 involves traversing all possible client selection scenarios and then selecting the scheme that minimizes the objective function. However, the complexity of this approach escalates rapidly with an increase in the total number of clients. Therefore, we introduce an efficient algorithm designed to address P4 in Algorithm 1. In this proposed algorithm, during each global iteration, clients with $I_{k}^{t}=P_{k}^{t}Z_{k}^{t}-V\mu q_{k}^{t}$ lower than 0 are included into the initial set $X_{0}^{t}$. Thereafter, considering that learning latency is determined by straggler clients, these $|X_{0}^{t}|$ clients are incorporated one by one into the auxiliary selection set $X_{a}^{t}$ in ascending order according to their total latency $\tau_{k}^{t}$, thereby generating $|X_{0}^{t}|$ auxiliary selection sets. Here |·| signifies the count of elements within the set. These $|X_{0}^{t}|$ auxiliary selection sets are subsequently accumulated in the client selection set $X^{t}$. We then compute the value of the objective function of P4 for each auxiliary selection set within $X^{t}$ and select the optimal auxiliary selection set ${\left(X_{a}^{t}\right)}^{*}$ that minimizes the objective function of P4. Utilizing our proposed algorithm, throughout each global iteration, only $|X_{0}^{t}|$ computations of the objective function are required to attain the optimal solution. Consequently, this represents a significantly lower complexity compared to the exhaustive traversal method.
**Algorithm 1** Client Selection Algorithm1:**Input:** $Z_{k}^{t}=0,\forall k,Y_{r}^{t}=0$2:Set $X_{0}^{t}=⌀,X_{a}^{t}=⌀,X^{t}=⌀$3:**for** k∈K **do**4:    Calculate $I_{k}^{t}=P_{k}^{t}Z_{k}^{t}-V\mu q_{k}^{t}$5:    **if** $I_{k}^{t}\leq 0$ **then**6:        $X_{0}^{t}=X_{0}^{t}\cup \left\{k\right\}$7:    **end if**8:**end for**9:Rank the clients in $X_{0}^{t}$ according to their $\tau_{k}^{t}$. Therefore we have $\tau_{1}^{t}\leq \tau_{2}^{t}\leq \ldots \leq \tau_{|X_{0}^{t}|}^{t}$10:**for** $x\in X_{0}^{t}$ **do**11:    Update $X_{a}^{t}=X_{a}^{t}\cup \left\{x\right\}$12:    Add $X^{t}$ to $X^{t}$, i.e., $X^{t}=X^{t}\cup \left\{X^{t}\right\}$13:    Calculate $J\left(X_{a}^{t}\right)=\sum_{k\in X_{a}^{t}}\left(P_{k}^{t}Z_{k}^{t}\right)+P_{r}^{t}Y_{r}^{t}+VU^{t}$14:**end for**15:Find ${\left(X_{a}^{t}\right)}^{*}=\arg\min_{X_{a}^{t}\in X^{t}}\left(J\left(X_{a}^{t}\right)\right)$16:Return ${\left(a^{t}\right)}^{*}$, where ${\left(a_{k}^{t}\right)}^{*}=1\left\{k\in {\left(X_{a}^{t}\right)}^{*}\right\},\forall k$

### 3.3. Analysis of the Proposed Optimization Scheme’s Optimality

Given the trade-off between minimizing the time-averaged cost and reducing power consumption violations, the analysis of the proposed optimization strategy’s optimality is provided herein.

**Theorem** **2.** *The average cost function satisfies:*

(28) $$\frac{1}{T}\sum_{t=0}^{T-1}E[U^{t}|\Theta^{t}]\leq \frac{C_{2}}{V}+\varphi^{*},$$

where $C_{2}\geq C_{1}+\sum_{k=1}^{K}Z_{k}^{t,\max}\max\left(P_{k}^{\max}-{\bar{P}}_{k}\right)+Y_{r}^{t,\max}\max\left(P_{r}^{\max}-{\bar{P}}_{r}\right)$, and $\varphi^{*}$ is the optimal solution of problem P1.

**Proof.**  The proof is given in Appendix B. □

**Theorem** **3.** Assume $E\left[U^{t}|\Theta^{t}\right]\geq \varphi^{\min}$. The power consumption of each client k and the server are bounded by $T{\bar{P}}_{k}+\sqrt{2TC_{2}+2TV\varphi^{*}-2TV\varphi^{\min}}$ and $T{\bar{P}}_{r}+\sqrt{2TC_{2}+2TV\varphi^{*}-2TV\varphi^{\min}}$, respectively.

**Proof.**  The proof is given in Appendix C. □

Theorem 2 elucidates that the discrepancy between the objective value yielded by the proposed algorithm and the original optimal value is less than or equal to O(1/V). This suggests that the cost determined by the proposed optimization scheme can approximate the original optimal value to an arbitrary degree through the augmentation of the control parameter *V*. In accordance with Theorem 3, the energy deficit queues of all clients and the server adhere to an upper limit of $O(\sqrt{V})$ at any iteration, a limit that escalates in accordance with the control parameter *V*. Nonetheless, an excessively large value of *V* may result in an unduly large upper boundary for the virtual power deficit queue backlog, which could lead to power consumption surpassing the power budget. In summary, the proposed algorithm delivers a $\left[O\left(1/V\right),O\left(\sqrt{V}\right)\right]$ trade-off between cost and power consumption, a balance that can be managed by adjusting the parameter *V*.

**Theorem** **4.** *Virtual queue of each client k and the server satisfies:*

(29) $$\lim_{T\to \infty }\frac{Z_{k}^{T}}{T}=0,\lim_{T\to \infty }\frac{Y_{r}^{T}}{T}=0.$$

**Proof.**  The proof is given in Appendix D. □

Theorem 4 indicates that the virtual power deficit queue backlog is bounded as the global iteration approaches infinity, i.e., all virtual queues remain mean rate stable across the FL iteration.

## 4. Experiment Result and Analysis

### 4.1. Experiment Settings

In the conducted experiment, FL was implemented using PyTorch, considering a system setup in which *K* clients are randomly positioned within a circular area of a 500 m radius with a central server. The path loss model is defined as $128.1+37.6\log_{10}i+\psi$, where *i* represents the distance between a client and the server in kilometers, while ψ is a Gaussian random variable exhibiting a variance of 8 dB. The total bandwidth, *B*, is set to 100 MHz, with the noise power spectral density $N_{0}=-174$ dBm/Hz.

The power used for uploading the local model is arbitrarily assigned between 10 and 20 dBm. The model size *s* is set as 1 Mbit. For all clients, the number of local iterations in each global iteration *m* is set to 1. The number of CPU cycles necessary for processing a sample per client is randomly distributed within the range of $\left[1,3\right]\times 10^{4}$ cycles/sample. Average power constraints are established at ${\bar{P}}_{k}=100$ mW and ${\bar{P}}_{r}=500$ mW. The decision parameter *V* is assigned the value of 10, with a justification provided later. The CPU frequency range of the clients and the central server, $f_{k}^{t}$ and $f_{r}^{t}$, span from 0.1 GHz to 2.5 GHz and from 0.1 GHz to 3.3 GHz, respectively. Furthermore, the capacitance coefficients for the clients and the server, the price parameter of the cost function, and the number of CPU cycles needed to perform a single summation are set to $\gamma_{1}=\gamma_{2}=10^{-28}$, $\mu =1.6\times 10^{-3}$, and $\phi =10^{6}$.

The MNIST dataset [21] was employed for the experiment, consisting of 60,000 training samples and 10,000 test samples with 10 label classes from 0 to 9. Each client’s local dataset was assembled by randomly selecting one or two label classes from the MNIST dataset with $d_{k}=100$ samples. A multi-layer perceptron (MLP) model with a single hidden layer containing 64 nodes was utilized, with ReLU as the activation function. The learning rate was set to 0.01, and the batch size was 10.

To demonstrate the advantage of our proposed algorithm, we introduce the following three algorithms as comparison benchmarks:

- Selected All: In this algorithm, all the clients are selected in each global iteration. The CPU frequency for both the clients and the central server is consistently set at their maximum values in every global iteration.
- Greedy: For a rational comparison with our proposed algorithm, the long-term average number of client selected per round is tuned to be consistent with that of our proposed algorithm in this comparative algorithm. As such, we establish a client selection latency threshold. Clients are subsequently chosen one by one in ascending order based on their individual total latency $\tau_{k}^{t}$ until the learning latency $\tau^{t}$ surpasses the preset client-selection latency threshold. Furthermore, with the prerequisite of adhering to the CPU frequency constraint, all participating clients and servers maintain a constant power level, identical to the long-term power constraint.
- Random: In this comparative algorithm, clients are randomly selected in each round. The number of clients selected is maintained at a constant value, which is equal to the average number per round in our proposed algorithm. Aside from this variation, all other configurations align with those of the Greedy algorithm.

### 4.2. Analysis of Experimental Results

**Figure 2:**
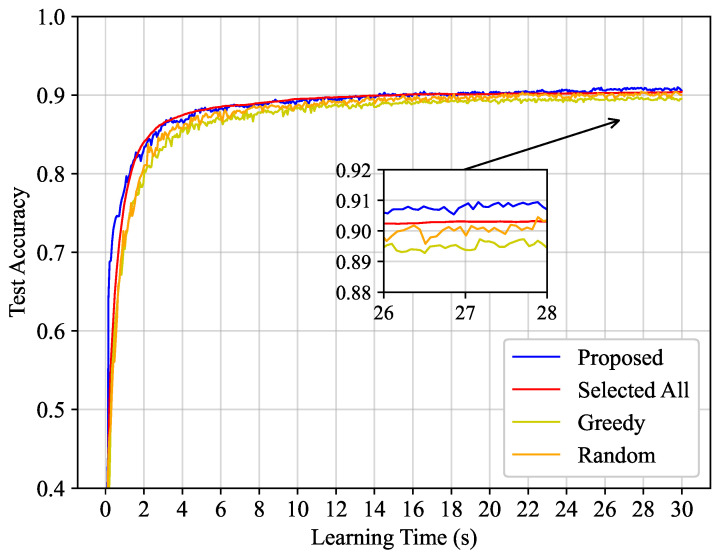
Test accuracy versus learning latency with the number of clients K=100.

Conceptually, reducing the time required for each global iteration and minimizing the impact of the non-IID nature on the model convergence speed enables the training model to reach a specific accuracy more rapidly within a given learning time. Figure 2 demonstrates how the test accuracy of our proposed algorithm and comparative algorithms varies with the learning time under the number of clients K=100. It is apparent that the proposed algorithm exhibits a performance almost equivalent to the Selected All algorithm in terms of convergence speed. Even though all the clients participate in each global iteration, fostering a swift convergence speed, the effects of the non-IID nature stemming from each client’s dataset cannot be negated, thereby undermining its performance. Conversely, in our proposed model, clients with more label classes in their dataset may inherently have a higher selection priority. Simultaneously, the mean rate stable properties of the virtual queue in the proposed algorithm ensure fairness for clients with fewer label classes. Our proposed model significantly outperforms both the Greedy and Random algorithms. In the Greedy algorithm, while the impact of straggler clients is mitigated, it does not address the influence of the non-IID nature. Since the presence of the non-IID nature and straggler clients are not taken into account, the convergence speed of the Random algorithm is impeded.

**Figure 3:**
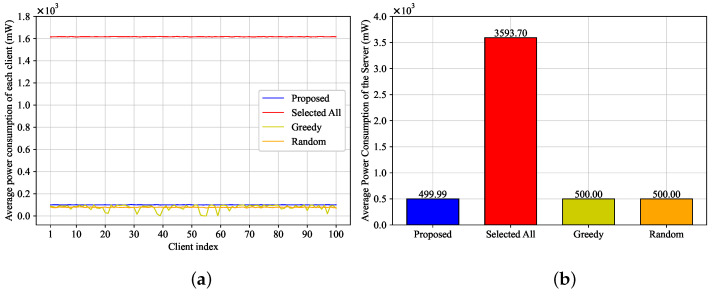
Average power consumption of each client and the server. (**a**) Each client. (**b**) Server.

Figure 3 demonstrates the corresponding average power consumption of the client side and the server side in each global iteration. The Selected All algorithm, when compared to other methods, exhibits substantially larger power consumption, primarily because it lacks a power constraint. However, due to the mean rate stable characteristic of the virtual queue, as demonstrated by Theorem 4, the power consumption under our proposed algorithm adheres to the long-term power constraint. Notably, the average power consumption under our proposed algorithm is approximately similar to that observed in the Greedy and Random algorithms.

**Figure 4:**
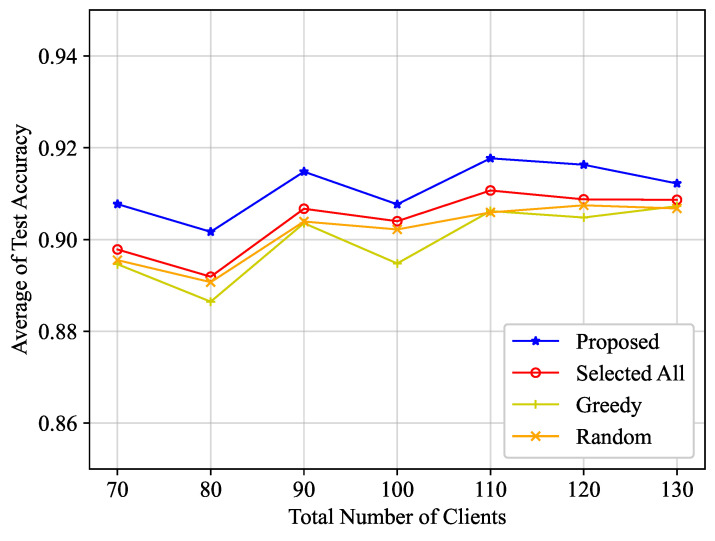
Average of test accuracy versus total number of clients.

To further validate the proposed optimization scheme, its performance is examined under a varying total number of clients *K*, as depicted in Figure 4 and Table 1. In the conducted experiment, we take into account the average test accuracy during the concluding half second when the learning time spans 30 seconds. Accompanying the increase in the total number of clients, the average number of clients selected per iteration also escalates, which, under normal circumstances, should enhance test accuracy. Nonetheless, the increase in the number of clients may cause a corresponding increment in each iteration’s training duration. As a consequence, the total iterations that can be carried out within a fixed time duration may decrease, thereby potentially reducing accuracy. Hence, the test accuracy does not bear a linear relationship with the total number of clients, which can be observed from Figure 4. Nevertheless, our proposed algorithm continues to surpass comparative algorithms, as it adeptly manages non-IID characteristics and straggler clients. Furthermore, our proposed algorithm exhibits a consistent ability to maintain low power consumption as the total number of clients increases. This finding aligns with our previous analysis, demonstrating that the virtual power deficit queues are mean rate stable in our proposed algorithm.

**Figure 5:**
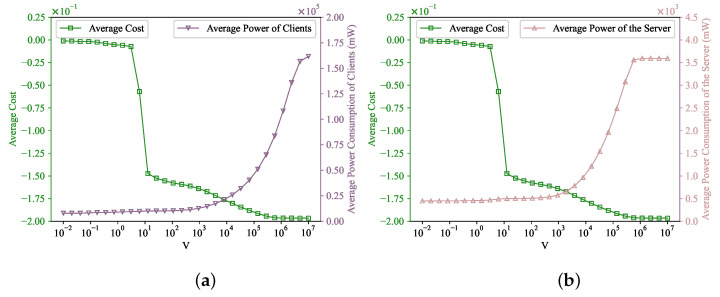
The impact of *V*. (**a**) Average power consumption of clients and average cost versus control parameter *V*. (**b**) Average power consumption of the server and average cost versus control parameter *V*.

**Figure 6:**
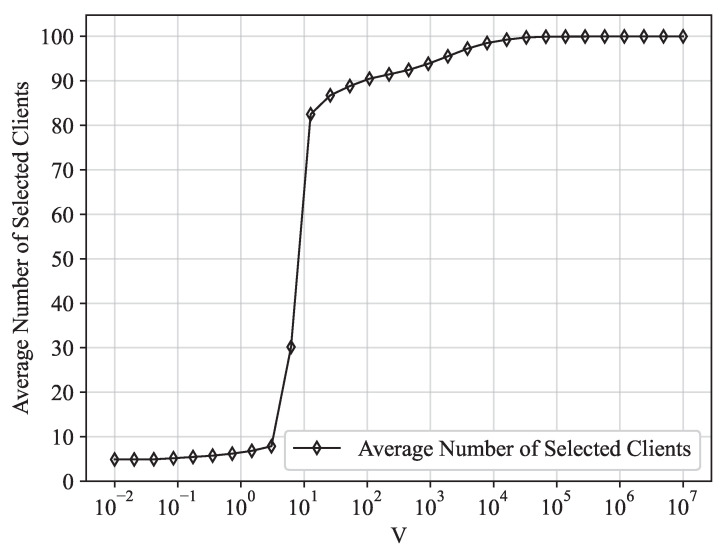
Average number of selected clients versus control parameter *V*.

Figure 5 depicts the variation in the average power consumption and the average cost in correlation with the control parameter *V* within our proposed optimization scheme. A clear observation is that the average cost experiences a decrease, while the average power consumption undergoes an increase with an escalating control parameter *V*. This aligns with the $\left[O\left(1/V\right),O\left(\sqrt{V}\right)\right]$ cost–power trade-off indicated by Theorems 2 and 3. Figure 6 illustrates the variation in the optimal average number of selected clients per iteration with the control parameter *V*. As previously stated, a control parameter value of V=10 was selected for the experiment. This choice was made because it yields an appropriate average number of selected clients to effectively address the accuracy degradation incited by non-IID characteristics, along with the low average cost and power consumption.

## 5. Discussion

In this paper, we explored a problem involving the selection of clients and the concurrent control of the CPU frequency for both the selected clients and the server within wireless FL networks. Lyapunov optimization theory was applied to transform the original problem into a per-iteration problem, which facilitated the design of an algorithm for problem resolution. Theoretical analysis offers performance guarantees, wherein controlling the parameter *V* empowers us to reduce cost while minimizing power consumption. Simulation results demonstrated that the proposed algorithm outperforms benchmark algorithms in terms of test accuracy by mitigating the impact of non-IID characteristics and straggler clients. By managing the virtual queues, the proposed algorithm was able to adhere to long-term power constraints. Furthermore, the simulation results verified that our proposed algorithm successfully realized the $\left[O\left(1/V\right),O\left(\sqrt{V}\right)\right]$ cost–power trade-off.

It is noteworthy that this study is currently confined to a simple star network topology. Expanding our analysis to encompass more intricate network structures such as hierarchical networks and multi-base station networks would undoubtedly enhance its applicability. Additionally, in practical wireless networks, client participation in learning can be affected by factors such as mobility, network congestion, or power availability fluctuations, potentially leading to client dropouts from the FL process and thereby impacting overall learning performance. Hence, the implications of client dropouts merit further investigation.

## Figures and Tables

**Table 1:** Average power consumption of clients and the server versus total number of clients.

Average Power Consumption	Total Number of Clients	Proposed	Selected All	Greedy	Random
clients	70	7016.21 mW	113,212.66 mW	5616.25 mW	5600.00 mW
	80	8020.03 mW	129,386.61 mW	6401.84 mW	6400.00 mW
	90	9033.43 mW	145,559.47 mW	7229.53 mW	7200.01 mW
	100	10,036.40 mW	161,733.26 mW	7743.76 mW	7800.00 mW
	110	11,030.69 mW	177,907.72 mW	8439.06 mW	8400.01 mW
	120	12,053.63 mW	194,080.37 mW	9336.41 mW	9300.01 mW
	130	13,047.53 mW	210,254.14 mW	9940.70 mW	9900.01 mW
server	70	499.86 mW	3593.70 mW	500.00 mW	500.00 mW
	80	499.94 mW	3593.70 mW	500.00 mW	500.00 mW
	90	499.94 mW	3593.70 mW	500.00 mW	500.00 mW
	100	499.99 mW	3593.70 mW	500.00 mW	500.00 mW
	110	500.03 mW	3593.70 mW	500.00 mW	500.00 mW
	120	500.04 mW	3593.70 mW	500.00 mW	500.00 mW
	130	500.24 mW	3593.70 mW	500.00 mW	500.00 mW

## Data Availability

Not applicable.

## Abbreviations

The following abbreviations are used in this manuscript:

FL	Federated learning
IoT	Internet of Things
non-IID	Non-independent and identically distributed
IID	Independent identically distributed
OFDMA	Orthogonal frequency-division multiple access
MLP	Multi-layer perceptron
ReLU	Rectified Linear Unit

## Appendix A

Since $\Delta (\Theta^{t})$ plays a crucial role in the proof, we first bound $\Delta (\Theta^{t})$. Plugging (17)–(19) into $\Delta (\Theta^{t})$, we have:

(A1) $$\begin{array}{c} \Delta \left(\Theta^{t}\right)=\frac{1}{2}E\left[\sum_{k=1}^{K}{\left(Z_{k}^{t+1}\right)}^{2}+{\left(Y_{r}^{t+1}\right)}^{2}-\sum_{k=1}^{K}{\left(Z_{k}^{t}\right)}^{2}-{\left(Y_{r}^{t}\right)}^{2}|\Theta^{t}\right]\\ =\frac{1}{2}E\left[\sum_{k=1}^{K}{\left\{\max\left\{P_{k}^{t}-{\bar{P}}_{k}+Z_{k}^{t},0\right\}\right\}}^{2}-\sum_{k=1}^{K}{\left(Z_{k}^{t}\right)}^{2}+{\left\{\max\left\{P_{r}^{t}-{\bar{P}}_{r}+Y_{r}^{t},0\right\}\right\}}^{2}-{\left(Y_{r}^{t}\right)}^{2}|\Theta^{t}\right]\\ \leq \frac{1}{2}E\left[\sum_{k=1}^{K}{\left(P_{k}^{t}-{\bar{P}}_{k}+Z_{k}^{t}\right)}^{2}-\sum_{k=1}^{K}{\left(Z_{k}^{t}\right)}^{2}+{\left(P_{r}^{t}-{\bar{P}}_{r}+Y_{r}^{t}\right)}^{2}-{\left(Y_{r}^{t}\right)}^{2}|\Theta^{t}\right]\\ \leq C_{1}+\sum_{k=1}^{K}Z_{k}^{t}E\left[P_{k}^{t}-{\bar{P}}_{k}|\Theta^{t}\right]+Y_{r}^{t}E\left[P_{r}^{t}-{\bar{P}}_{r}|\Theta^{t}\right], \end{array}$$

where the first inequality is due to ${\left\{\max\left\{a,0\right\}\right\}}^{2}\leq a^{2}$. Thus, we have:

(A2) $$\Delta \left(\Theta^{t}\right)+VE\left[U^{t}|\Theta^{t}\right]\leq C_{1}+\sum_{k=1}^{K}Z_{k}^{t}E\left[P_{k}^{t}-{\bar{P}}_{k}|\Theta^{t}\right]+Y_{r}^{t}E\left[P_{r}^{t}-{\bar{P}}_{r}|\Theta^{t}\right]+VE\left[U^{t}|\Theta^{t}\right],$$

This concludes the proof.

## Appendix B

According to Appendix A, we have:

(A3) $$\begin{array}{c} \Delta \left(\Theta^{t}\right)+VE\left[U^{t}|\Theta^{t}\right]\\ \leq C_{1}+\sum_{k=1}^{K}Z_{k}^{t,\max}\max\left(E\left[P_{k}^{\max}-{\bar{P}}_{k}|\Theta^{t}\right]\right)+Y_{r}^{t,\max}\max\left(E\left[P_{r}^{\max}-{\bar{P}}_{r}|\Theta^{t}\right]\right)+V\varphi^{*}\\ \leq C_{2}+V\varphi^{*} \end{array}$$

Summing (A3) over *T* global iterations, we obtain:

(A4) $$\sum_{t=0}^{T-1}VE[U^{t}|\Theta^{t}]\leq TC_{2}+TV\varphi^{*}-\sum_{t=0}^{T-1}\Delta (\Theta^{t}).$$

Next we sum $\Delta (\Theta^{t})$ over *T* global iterations. The following formula can be derived from (20):

(A5) $$\sum_{t=0}^{T-1}\Delta (\Theta^{t})=L\left(\Theta^{T}\right)-L\left(\Theta^{0}\right).$$

Plugging (A5) into (A4), we have:

(A6) $$\sum_{t=0}^{T-1}VE[U^{t}|\Theta^{t}]\leq TC_{2}+TV\varphi^{*}-L\left(\Theta^{T}\right)+L\left(\Theta^{0}\right).$$

Dividing by TV, using the fact that $L(\Theta^{T})\geq 0$, $L(\Theta^{0})=0$, we have:

(A7) $$\frac{1}{T}\sum_{t=0}^{T-1}E[U^{t}|\Theta^{t}]\leq \frac{C_{2}}{V}+\varphi^{*}.$$

This concludes the proof.

## Appendix C

Rearranging (A6), we obtain:

(A8) $$L\left(\Theta^{T}\right)-L\left(\Theta^{0}\right)\leq TC_{2}+TV\varphi^{*}-\sum_{t=0}^{T-1}VE[U^{t}|\Theta^{t}].$$

Substituting $E\left[U^{t}|\Theta^{t}\right]\geq \varphi^{\min}$ into (A8), we have:

(A9) $$L\left(\Theta^{T}\right)\leq TC_{2}+TV\varphi^{*}-TV\varphi^{\min}.$$

Due to (19), we obtain:

(A10) $${\left(Z_{k}^{T}\right)}^{2}+{\left(Y_{r}^{T}\right)}^{2}\leq \sum_{k=1}^{K}{\left(Z_{k}^{T}\right)}^{2}+{\left(Y_{r}^{T}\right)}^{2}\leq 2TC_{2}+2TV\varphi^{*}-2TV\varphi^{\min}.$$

Thus, we have:

(A11) $${\left(Z_{k}^{T}\right)}^{2}\leq 2TC_{2}+2TV\varphi^{*}-2TV\varphi^{\min},{\left(Y_{r}^{T}\right)}^{2}\leq 2TC_{2}+2TV\varphi^{*}-2TV\varphi^{\min}.$$

Next we bound the virtual power deficit queues. From (17) and (18), we have:

(A12) $$Z_{k}^{t+1}=Z_{k}^{t}-\min\left\{{\bar{P}}_{k}-P_{k}^{t},Z_{k}^{t}\right\},Y_{r}^{t+1}=Y_{r}^{t}-\min\left\{{\bar{P}}_{r}-P_{r}^{t},Y_{r}^{t}\right\}.$$

Rearranging terms and summing both sides over *t* global iterations, we have:

(A13) $$Z_{k}^{t}-Z_{k}^{0}=-\sum_{\tau =0}^{t-1}\min\{{\bar{P}}_{k}-P_{k}^{\tau },Z_{k}^{t}\}\geq \sum_{\tau =0}^{t-1}\left(P_{k}^{\tau }-{\bar{P}}_{k}\right)=\sum_{\tau =0}^{t-1}P_{k}^{\tau }-t{\bar{P}}_{k},$$

(A14) $$Y_{r}^{t}-Y_{r}^{0}=-\sum_{\tau =0}^{t-1}\min\{{\bar{P}}_{r}-P_{r}^{\tau },Y_{k}^{t}\}\geq \sum_{\tau =0}^{t-1}\left(P_{r}^{\tau }-{\bar{P}}_{r}\right)=\sum_{\tau =0}^{t-1}P_{r}^{\tau }-t{\bar{P}}_{r}.$$

Thus, we have:

(A15) $$Z_{k}^{T}\geq \sum_{\tau =0}^{T-1}P_{k}^{\tau }-T{\bar{P}}_{k},Y_{r}^{T}\geq \sum_{\tau =0}^{T-1}P_{r}^{\tau }-T{\bar{P}}_{r}.$$

Thus far, we have bounded the virtual power deficit queues. Plugging (A15) into (A11), we have:

(A16) $$\sum_{\tau =0}^{T-1}P_{k}^{\tau }\leq T{\bar{P}}_{k}+\sqrt{2TC_{2}+2TV\varphi^{*}-2TV\varphi^{\min}},$$

(A17) $$\sum_{\tau =0}^{T-1}P_{r}^{\tau }\leq T{\bar{P}}_{r}+\sqrt{2TC_{2}+2TV\varphi^{*}-2TV\varphi^{\min}}.$$

This concludes the proof.

## Appendix D

Rearranging terms of (A11), we have:

(A18) $$Z_{k}^{T}\leq \sqrt{2TC_{2}+2TV\varphi^{*}-2TV\varphi^{\min}},Y_{r}^{T}\leq \sqrt{2TC_{2}+2TV\varphi^{*}-2TV\varphi^{\min}}.$$

Dividing by *T* and taking limits of both sides, we have:

(A19) $$\lim_{T\to \infty }\frac{Z_{k}^{T}}{T}=0,\lim_{T\to \infty }\frac{Y_{r}^{T}}{T}=0.$$

This concludes the proof.
